# Assessing Total Hip Arthroplasty Outcomes and Generating an Orthopedic Research Outcome Database via a Natural Language Processing Pipeline: Development and Validation Study

**DOI:** 10.2196/64705

**Published:** 2025-03-12

**Authors:** Nicholas H Mast, Clara L. Oeste, Dries Hens

**Affiliations:** 1 Hip and Pelvis Institute, 2250 Hayes St # 208, San Francisco, CA, 94117, United States, 1 415-530-5330; 2 LynxCare Inc, Leuven, Belgium

**Keywords:** total hip arthroplasty, THA, direct anterior approach, electronic health records, EHR, natural language processing, NLP, complication rate, single-surgeon registry, hip arthroplasty, orthopedic, validation, surgeon, outpatient visits, hospitalizations, surgery

## Abstract

**Background:**

Processing data from electronic health records (EHRs) to build research-grade databases is a lengthy and expensive process. Modern arthroplasty practice commonly uses multiple sites of care, including clinics and ambulatory care centers. However, most private data systems prevent obtaining usable insights for clinical practice.

**Objective:**

This study aims to create an automated natural language processing (NLP) pipeline for extracting clinical concepts from EHRs related to orthopedic outpatient visits, hospitalizations, and surgeries in a multicenter, single-surgeon practice. The pipeline was also used to assess therapies and complications after total hip arthroplasty (THA).

**Methods:**

EHRs of 1290 patients undergoing primary THA from January 1, 2012 to December 31, 2019 (operated and followed by the same surgeon) were processed using artificial intelligence (AI)–based models (NLP and machine learning). In addition, 3 independent medical reviewers generated a gold standard using 100 randomly selected EHRs. The algorithm processed the entire database from different EHR systems, generating an aggregated clinical data warehouse. An additional manual control arm was used for data quality control.

**Results:**

The algorithm was as accurate as human reviewers (0.95 vs 0.94; *P=*.01), achieving a database-wide average *F*_1_-score of 0.92 (SD 0.09; range 0.67‐0.99), validating its use as an automated data extraction tool. During the first year after direct anterior THA, 92.1% (1188/1290) of our population had a complication-free recovery. In 7.9% (102/1290) of cases where surgery or recovery was not uneventful, lateral femoral cutaneous nerve sensitivity (47/1290, 3.6%), intraoperative fractures (13/1290, 1%), and hematoma (9/1290, 0.7%) were the most common complications.

**Conclusions:**

Algorithm evaluation of this dataset accurately represented key clinical information swiftly, compared with human reviewers. This technology may provide substantial value for future surgeon practice and patient counseling. Furthermore, the low early complication rate of direct anterior THA in this surgeon’s hands was supported by the dataset, which included data from all treated patients in a multicenter practice.

## Introduction

Total hip arthroplasty (THA) is one of the most effective orthopedic procedures [1]. The number of THAs is projected to rise, along with its impact on health care resources [2]. However, more government funding requires efficient quality control and accountability. Quality monitoring is expected to have a bigger impact on orthopedic practices and on medicine as a whole in the near future. Unfortunately, many surgeons working in nonacademic settings frequently lack the resources and financing needed to properly evaluate the quality of patient care they provide. The broad adoption of electronic health records (EHRs) simplifies access to vast amounts of medical data and facilitates data analysis.

Despite how inefficient and time-consuming manual review is, it remains the gold standard for reviewing medical information. Research has shown that systematic reporting of postoperative orthopedic surgical adverse events is infrequent [3], despite the low complication rates associated with THA. Monitoring the standard of care after THA is still challenging, since evaluating sufficient records in a timely manner is a complex task. Using natural language processing (NLP) technology, we developed a system that can extract relevant postoperative problems from unstructured EHR data [4]. We questioned whether such an AI-supported approach might be used to provide precise, continuing feedback on the quality of care provided after THA in a high-volume, nonacademic clinical setting. We assumed that a computer algorithm would perform at least as well as a human reviewer, which is considered the industry standard.

The primary goal of this study was, therefore, to design and develop an automated NLP pipeline for extracting clinical concepts from reports on outpatients, hospitalizations, and surgeries from a single surgeon, multicenter practice. Using the pipeline, the secondary goal was to examine and evaluate therapies and complications following THA.

## Methods

### Ethical Considerations

Patients were invited to participate in the research on the direct anterior approach (DAA) of hip replacement. Informed consent for the use of patient data in this retrospective study was obtained as part of the institutional review board (IRB) protocol (WCG IRB Protocol 20225993). Patients undergoing surgery were provided with a consent form at the time of intake, which was signed before the procedure. Patients who continued to be followed up subsequently signed consent forms that were applicable retrospectively. Data were anonymized before analysis.

### Patient Selection

Data from 1290 patients undergoing 1304 primary DAA THAs using a Hana Orthopedic Surgery Table (Mizuho OSI), with the same surgical team and implants. Data was collected from January 1, 2012 to December 31, 2019, and analyzed retrospectively. Exclusion criteria included age under 18 years, failure to provide informed consent, and revision surgery.

### Follow-Up Schedule

Patients were followed preoperatively and postoperatively at 2 weeks, 6 weeks, 3 months (on a per-case basis), and 1 year. The algorithm screened EHRs for key sentences, words, and clinical definitions that were compiled by the surgical team.

### Adverse Event Classification

A list of 19 complications based on the study of the Hip Society THA Complications Workgroup was compiled [5]. A separate category was added for lower back pain, sciatica, or both. Treatment was categorized as either conservative or interventional.

### EHR Data Structure and Documentation Variability

The study used EHR systems that capture all patient interactions across the health system. Documentation varied by note type: operative reports were dictated using a standardized format, with intraoperative complications documented in a dedicated subsection. Radiographic reports, however, were dictated by multiple radiologists without a uniform structure. Complications were often identifiable through multiple associated notes across different document types. Implant records were dictated directly from intraoperative purchasing logs, ensuring high reliability. The NLP algorithm flagged typographical errors in dictated notes that human reviewers overlooked.

### Gold Standard Adjudication Process

To ensure consistency in the gold standard corpus used for training the algorithm, all discrepancies among reviewers were resolved through senior adjudication. When ambiguities were identified in a case, they were escalated to the senior author, who conducted a thorough review of the full case file. Final determinations were made based on clinical expertise and consensus, ensuring a standardized and reliable reference dataset.

### NLP Pipeline Development and Validation

The NLP pipeline was developed and validated in 3 phases, as described by Van de Meulebroucke et al [4]. First, a training set of 50 EHRs was annotated to generate relevant input, since a sample size of 50 EHRs was previously found sufficient to achieve accuracy over 90% [4]. Second, an independent gold standard was generated through manual review by 3 independent surgeons of a derivation cohort of 100 EHRs. Third, the entire dataset of 1290 patients was manually reviewed to calculate the algorithm’s recall, precision, and *F*_1_-score. Patient records were analyzed using standard procedures of each center’s EHR system by a manual reviewer.

The pipeline uses a text mining engine based on machine learning and NLP to extract important concepts from EHRs. The NLP pipeline incorporates several preprocessing steps, including language detection, sentence segmentation, and tokenization, to prepare unstructured EHR text for analysis. The core architecture is based on a deep learning model fine-tuned on large-scale clinical datasets and adapted for domain-specific tasks in orthopedics. Named entity recognition identifies relevant medical concepts, relation extraction determines associations between entities, and attribute extraction captures features such as negations and temporal qualifiers. Processed outputs were mapped to Unified Medical Language System (UMLS) Concept Unique Identifiers (CUIs), which includes standard terminologies like the Systematized Nomenclature of Medicine-Clinical Terms (SNOMED-CT). Mapping was performed by a probabilistic entity-linking algorithm that integrates semantic ranking and term preferences, ensuring precise and standardized representation. In addition, the processed data were standardized using the Observational Medical Outcomes Partnership Common Data Model, which harmonizes structured and unstructured data from different EHR systems into a unified format. This approach enables the efficient processing of unstructured clinical text and supports the accurate extraction and mapping of clinical concepts.

**Figure 1.**
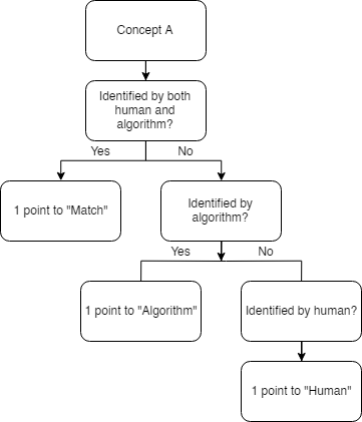
Scoring system flowchart for concept identification by human versus algorithm.

Complications and their respective treatment, along with other clinical features, were collected into a database and standardized using UMLS CUIs. The NLP tool mined variables from the full set of source data within EHRs, including, but not limited to, clinical notes, primary care records, radiology reports, laboratory results, discharge summaries, and surgical documentation. The full list of study variables is shown in Multimedia Appendix 1. To assess accuracy, a scoring system was established such that one point was given to each correct clinical concept, extracted by the algorithm or the human reviewer (Figure 1). Correct clinical concepts referred to those matching the gold standard. Precision, recall, and *F*_1_-score were used as statistical parameters to reflect the performance of the algorithm, as calculated with R (R Foundation for Statistical Computing) statistical programming (Table 1).

**Table 1.** Statistical parameters for algorithm performance.

	Definition	Calculation
Accuracy	Percentage (%) of relevant concepts extracted by method A/B.	Number of concepts÷total number of concepts expected to be extracted
Recall or sensitivity	Ratio between all identified concepts and all existing concepts in a given text (0‐1).	True positives÷(true positives+false negatives)
Precision	Ratio between all adequately identified concepts and all identified concepts (0‐1).	True positives÷(true positives+false positives)
*F*_1_-score	Harmonic mean of recall and precision (0‐1).	2×(recall×precision)÷(recall+precision)

### Statistical Analysis

For categorical values, the number of patients (n) and relative proportions (%) are shown.

Checks for accuracy, recall, and precision involved measuring model outcomes against a gold standard created by human annotators. The *F*_1_-score assesses the trade-off between precision and recall by calculating their harmonic mean (Table 1).

## Results

### Performance of the NLP Pipeline

Table 2 compares the accuracy of human reviewers to an algorithm on various aspects of 100 patient records. Overall, the algorithm was slightly more accurate than human reviewers, with an accuracy of 0.952 compared with 0.945 for human reviewers, a difference of 0.007 (*P=*.01). The most notable differences in accuracy between the algorithm and human reviewers were on the variables of weight (0.24 difference in accuracy), revision (0.06 difference), and offset (0.02 difference), all of which were detected more accurately by the algorithm. The algorithm was less accurate on variables such as cup size, prosthetic loosening, and heterotopic ossification grade, though with small differences in accuracy ranging from −0.06 to 0.04.

In order to further assess the quality of data extracted by the algorithm, we calculated statistical parameters such as recall, precision, and *F*_1_-score of the different variables detected in the study, as defined in Table 1. The results shown in Table 3 reflect that for most variables, the recall and *F*_1_-score are high, indicating that the model is able to correctly identify the presence of the clinical variables in the data. The average values for all the variables show an overall high performance of the model.

**Table 2.** Human versus algorithm accuracy on a randomly selected group of 100 patients.

	Human reviewer accuracy	Algorithm accuracy	Delta
Antibiotics	1	1	0
Blood loss	1	0.98	−0.02
Cup size	0.98	0.92	−0.06
Date of procedure	0.98	0.98	0
Dislocation	0.96	0.94	−0.02
Fracture	0.98	0.96	−0.02
Heterotopic ossification grade	0.9	0.92	0.02
Offset	1	0.98	−0.02
Pain killer	0.96	0.96	0
Primary arthrosis	0.98	0.98	0
Prosthesis manufacturer	0.94	0.96	0.02
Prosthetic loosening	0.96	0.92	−0.04
Psoas tendinopathy	1	0.96	−0.04
Range of motion	0.86	0.88	0.02
Revision	0.9	0.96	0.06
Steel type	0.9	0.92	0.02
Surgery duration	1	0.98	−0.02
Thromboembolism	1	0.98	−0.02
Weight	0.64	0.88	0.24
Wound infection	0.96	0.98	0.02
Total	0.945	0.952	0.007

**Table 3.** Recall, precision, and *F*_1_-scores for entire dataset of 1290 cases across all data points.

	Recall	Precision	*F*_1_-score
Superficial wound infection	1	0.88	0.94
Heterotopic ossification	0.92	0.95	0.93
Lower back pain	1	0.98	0.99
Peri-prosthetic Infection	0.98	0.95	0.96
Wound infection	0.95	0.82	0.88
Dislocation	1	0.99	0.99
Revision	1	0.97	0.98
Intra-operative fracture	1	0.99	0.99
Post-operative fracture	1	0.89	0.94
Bleeding	0.95	0.65	0.77
Prosthetic loosening	1	0.98	0.99
Pain	0.97	0.88	0.92
Nerve injury	0.89	0.92	0.90
Psoas burden	0.99	0.99	0.99
Sciatica	0.75	0.88	0.81
Vascular systems injury	1	0.5	0.67
Average	0.96	0.89	0.92

### Complications and Interventions of DAA THA

The mean age of the patients included in the dataset was 63.5 (SD 9.6) years and 57.4% (740/1290) were women. The complications during the first year after direct anterior THA in these patients are shown in Table 4, as well as the conservative or interventional treatment of these clinical outcomes.

Soft tissue complications were the most common type of complication observed in patients undergoing THA. The most common type of soft tissue complication was sensory deficit (47/1290, 3.6%), followed by hematoma (9/1290, 0.7%) and superficial wound infection (3/1290, 0.2%). No incidents of bleeding, prolonged wound drainage, motor nerve deficit, vascular injury, or sciatica were reported. Regarding mechanical complications, periprosthetic fractures occurred in 1.4% of cases (18/1290; 13 intraoperatively and 5 postoperatively), and instability occurred in 0.5% (7/1290) of cases. Implant fracture, cup-liner dislocation and osteolysis were not reported.

Table 5 shows interventions in the 1-year postoperative period. The most common interventions were intraoperative (cerclage and wiring), which account for 1% (n=13) of cases. Readmission was detected in 0.9% (n=12) of patients. Other interventions such as aspiration, wound debridement, closed reduction, osteosynthesis or cerclage, and revision, each account for less than 1% of cases.

**Table 4.** Complications in the 1-year postoperative period (n=1290).

Complication subgroup	Total, n (%)	Conservative treatment, n (%)	Interventional treatment, n (%)
Soft tissue complications: wounds			
Bleeding	0 (0)	0 (0)	0 (0)
Hematoma	9 (0.7)	6 (0.5)	3 (0.2)
Prolonged wound drainage	1 (0.1)	0 (0)	1 (0.1)
Soft tissue complications: infection			
Superficial wound infection	3 (0.2)	3 (0.2)	0 (0)
Deep PJI^a^	3 (0.2)	0 (0)	3 (0.2)
Soft tissue complications: neural deficit			
Motor nerve deficit	0 (0)	0 (0)	0 (0)
Transient sensory deficit (LFCN^b^ sensitivity)	47 (3.6)	47 (3.6)	0 (0)
Soft tissue complications: lower back pain			
Lumbago	6 (0.5)	6 (0.5)	0 (0)
Sciatica	1 (0.1)	1 (0.1)	0 (0)
Soft tissue complications: other			
Vascular Injury	0 (0)	0 (0)	0 (0)
Heterotopic Ossification	2 (0.2)	2 (0.2)	0 (0)
Thromboembolic disease	3 (0.2)	3 (0.2)	0 (0)
Mechanical complications: periprosthetic fracture			
Intraoperative	13 (1)	0 (0)	13 (1)
Postoperative	5 (0.4)	0 (0)	5 (0.4)
Mechanical complications: other			
Instability	7 (0.5)	3 (0.2)	4 (0.3)
Implant fracture	0 (0)	0 (0)	0 (0)
Loosening	2 (0.2)	0 (0)	2 (0.2)
Cup-liner dislocation	0 (0)	0 (0)	0 (0)
Bearing surface wear	1 (0.1)	0 (0)	1 (0.1)
Osteolysis	0 (0)	0 (0)	0 (0)

a PJI: prosthetic joint infection.

b LFCN: lateral femoral cutaneous nerve.

**Table 5.** Interventions in the 1-year post-operative period.

Intervention	Values (n=1290), n (%)
Intraoperative	
Cerclage and wiring	13 (1)
Outpatient	
Infiltration	0 (0)
Aspiration	3 (0.2)
Reoperation	
Wound debridement	2 (0.2)
Closed reduction	2 (0.2)
Osteosynthesis or cerclage	2 (0.2)
Other	
Revision	9 (0.7)
Readmission	12 (0.9)

## Discussion

### Principal Findings

This study reflects the use of AI techniques to streamline and improve data capture from EHRs, in line with the current need for innovative solutions to obtain meaningful information from existing data sources in the field of orthopedics [6]. AI-supported automation, substantiated in the first step by directly comparing it with a manual review of a random sample of patients, showed no statistical differences in accuracy across all compared clinical concepts. Subsequently, application of the algorithm to the entire dataset generated very high *F*_1_-scores, therefore, validating its ability to detect positive results in our cohort with a high degree of precision and recall. This allowed a single-surgeon registry to be populated from existing health data within EHRs in a very short period of time. The NLP pipeline handled documentation variability using named entity recognition, probabilistic entity linking, and attribute extraction, allowing synonym recognition and standardization to SNOMED-CT and UMLS CUIs. This data-driven approach ensured robust identification of complications across different documentation sources. Furthermore, the methodology deployed in this study allows for inclusion of data from different software sources, which can be a limitation of single-surgeon registries that must be populated from diverse clinics or ambulatory care centers. The resulting multicenter, single-surgeon database is comparable in its granularity to single-system registries populated with conventional methods, circumventing the complexity of a system that is becoming increasingly fragmented.

The methodology that was previously described in a smaller cohort of Dutch patients is validated here in a multicenter, US-based practice, screening records in English from 1290 patients over an 8-year period at high accuracy [4]. It also demonstrates its usefulness in the nonacademic setting, compared with previous studies at academic centers [7,8]. Analysis of such a large cohort from a single surgeon permitted categorization of frequent concepts without needing to account for the confounding variables of different techniques and complication rates of multiple surgeons [9].

Our dataset split balances annotation feasibility and model performance, following previous clinical NLP studies. Small, high-quality training sets have been shown to be effective in constrained domains, particularly when leveraging structured reporting patterns [10]. The chosen test set size aligns with established practices in clinical NLP research [11]. Instead of relying solely on predefined training-validation-test ratios, we incorporated post hoc validation on a large dataset to assess generalizability, a common strategy in clinical text mining [12,13].

In addition, clinical insight was also gained regarding the frequency and management of complications over a 1-year period. While the study highlights the effectiveness of NLP tools in single surgeon practices, extensive datasets can greatly benefit from its capabilities. NLP tools offer versatility in both big data settings and smaller subsets, enabling comprehensive analysis across various practice settings and facilitating evaluation of outcomes, for example, relative to regional averages or previously published work.

Ultimately, concept detection and extraction of the pipeline demonstrated accuracy on par with human reviewers at a fraction of the cost and time required for manual chart review. Validation is a necessary step for algorithm use that benefits from physicians’ feedback through annotation. This input is necessary to test algorithm performance, to calibrate the algorithm’s rates of false positives and false negatives, and to examine if clinical use of algorithms leads to better research insights and health care services [14]. The ease and high degree of fidelity of concept extraction by the algorithm in this study facilitated longitudinal evaluation of clinical complications, and importantly, also precluded the need to manually filter through patient charts, which is time-consuming [15].

### Comparison With Previous Work

The representativeness of the methodology in this study is hence reflected by the frequency of the complications, which falls within the lower range of previously described cohorts [16]. For instance, a frequency of 1% of periprosthetic fractures was detected, which was the most common intraoperative complication in this study, and was found to be 2.1%, 1.6%, and 1.1% in other cohorts [4,9,17]. Other similar comparisons show that revisions were detected in 0.7% (9/1290) of patients in our study vs 2.1% (5/239) and 1.6% (3/187), and prosthetic joint infection, at 0.2% (3/1290) in our study, appeared in 1.3% (3/239) and 1.6% (3/187) of patients in the aforementioned studies [9,17]. In a prospective comparison of DAA to posterior approach THA (43 DAA THAs in total), 2.3% (1/43) had hematoma vs 0.7% (9/1290) in our study, 2.3% (1/43) presented heterotopic ossification vs 0.2% (3/1290) shown here, and neither study detected dislocations [18]. Therefore, the results obtained using the validated, NLP-driven methodology shown here also highlight the quality of practice of the surgeon, for the complications described in this study fall within the lower range of those previously described in the literature [9,16-20].

### Limitations

As is the case in real-world evidence studies, there are certain limitations to consider for this study, such as missing values for certain variables. Regarding rare concepts such as bleeding, the scarcity of terms found in the EHRs may hinder algorithm training. The algorithm solely focused on patients undergoing DAA THA, which is the method used by the surgeon, but its validity could be further strengthened by expanding the analyses to other populations. In addition, the quality of records kept at each site determines the quality of algorithm training, highlighting the need for ongoing validation processes as the database expands. Missing structured data were treated as true absences and were not imputed. For unstructured text, missing values could reflect a lack of documentation or true absences. Manual review helped assess whether missing information stemmed from documentation gaps or NLP constraints. While a single-surgeon dataset ensures consistency in surgical technique and documentation style, it may limit generalizability to other surgical practices. However, the inclusion of multisource EHR data and independent validation by multiple reviewers helps mitigate this potential bias.

### Conclusions

In this study, the NLP pipeline proved highly effective in improving data capture and enhancing clinical outcomes analysis following DAA THA, overcoming constraints linked to single-surgeon registries, which typically require data aggregation from multiple care sites. It holds significant potential for future orthopedic studies, providing accurate and cost-effective insights. In addition, the dataset supports the low early complication rate of direct anterior THA in this surgeon’s multicenter practice.

## Supplementary material

10.2196/64705 Multimedia Appendix 1 Data sources, categories, and concept unique identifiers of all clinical data points.

## Abbreviations

AI: artificial intelligence
CUI: Concept Unique Identifier
DAA: direct anterior approach
EHR: electronic health record
NLP: natural language processing
SNOMED-CT: Systematized Nomenclature of Medicine-Clinical Terms
THA: total hip arthroplasty
UMLS: Unified Medical Language System
