# Supplementary material for: Assessing Total Hip Arthroplasty Outcomes and Generating an Orthopedic Research Outcome Database via a Natural Language Processing Pipeline: Development and Validation Study
Source: JMIR Med Inform. 2025 Mar 12;13:e64705. doi: 10.2196/64705 (PMC11922490; doi:10.2196/64705)
Supplement: Multimedia Appendix 1 [file medinform-v13-e64705-s001.docx]

Table S1: Data sources, categories, and concept unique identifiers of all clinical data points

|  | **Data source** | **Semantic category** | **Category** | **Concept unique identifier (CUI)** |
| --- | --- | --- | --- | --- |
|  |  |  |  |  |
| Congenital hip joint deformity | Consultation note | Problem | Co-existing disorder | C0265615 |
| Depression | Consultation note | Problem | Co-existing disorder | C0011581 |
| Diabetes Mellitus | Consultation note | Problem | Co-existing disorder | C0011849 |
| Heart Diseases | Consultation note | Problem | Co-existing disorder | C0018799 |
| Hypertension | Consultation note | Problem | Co-existing disorder | C0020538 |
| Kidney Diseases | Consultation note | Problem | Co-existing disorder | C0022658 |
| Liver Diseases | Consultation note | Problem | Co-existing disorder | C0023895 |
| Lung Diseases | Consultation note | Problem | Co-existing disorder | C0024115 |
| Neoplastic disease | Consultation note | Problem | Co-existing disorder | C1882062 |
| Nervous system disorder | Consultation note | Problem | Co-existing disorder | C0027765 |
| Pain of bilateral lower limbs co-occurrent and due to ischemia | Consultation note | Problem | Co-existing disorder | C4303597 |
| Rheumatoid arthritis | Consultation note | Problem | Co-existing disorder | C0003873 |
| Spondylarthritis | Consultation note | Problem | Co-existing disorder | C0949690 |
| Stroke / cerebrovascular accident (CVA) | Consultation note | Problem | Co-existing disorder | C0038454 |
| Abductor pain | Consultation note | Problem | Complications | C0409352 |
| Adductor pain | Consultation note | Problem | Complications | C0409351 |
| ALVAL | Consultation note | Problem | Complications | LC0040063 |
| Bleeding | Consultation note | Problem | Complications | C0019080 |
| Cement-related hypotension | Consultation note | Problem | Complications | C0348663 |
| Gluteal pain | Consultation note | Problem | Complications | LC0011821 |
| HAPU/Pressure ulcer | Consultation note | Problem | Complications | C0011127 |
| Heterotopic Ossification | Consultation note | Problem | Complications | C0029396 |
| Hip Dislocation | Consultation note | Problem | Complications | C0019554 |
| Hip instability | Consultation note | Measurement | Complications | C0427257 |
| Hypotension during surgery | Consultation note | Problem | Complications | C4517362 |
| Implant failure | Consultation note | Problem | Complications | C4534315 |
| Intra-operative fracture | Consultation note | Problem | Complications | C0436007 |
| Ischialgy (sciatica) | Consultation note | Problem | Complications | C0036396 |
| Leg Length Inequality | Consultation note | Problem | Complications | C0264156 |
| Low back pain | Consultation note | Problem | Complications | C0024031 |
| Lytic lesion | Consultation note | Problem | Complications | C0221204 |
| Nerve injury | Consultation note | Problem | Complications | C0161479 |
| Neural deficit | Consultation note | Measurement | Complications | C0521654 |
| Overall complication | Consultation note | Problem | Complications | C0009566 |
| Pain at night | Consultation note | Problem | Complications | C0234255 |
| Periprosthetic Fractures | Consultation note | Problem | Complications | C2609162 |
| Periprosthetic Infection | Consultation note | Problem | Complications | C0349726 |
| Post-operative complication | Consultation note | Problem | Complications | C0032787 |
| Prosthesis Loosening | Consultation note | Problem | Complications | C0033587 |
| Psoas burden | Consultation note | Problem | Complications | C0277849 |
| Pulmonary embolism (PE) | Consultation note | Problem | Complications | C0034065 |
| Rectus femoris pain | Consultation note | Problem | Complications | C0410269 |
| Tensor fascia pain | Consultation note | Problem | Complications | C4285861 |
| Thromboembolism | Consultation note | Problem | Complications | C0040038 |
| Thrombophlebitis | Consultation note | Problem | Complications | C0040046 |
| Vascular System Injuries | Consultation note | Problem | Complications | C0178324 |
| Wear of articular bearing surface of joint prosthesis of hip | Consultation note | Problem | Complications | C2711887 |
| Wound infection | Consultation note | Problem | Complications | C0043241 |
| Wound leakage | Consultation note | Problem | Complications | C0151692 |
| BMI | Consultation note | Measurement | Demographic factors | C1305855 |
| Height | Consultation note | Measurement | Demographic factors | C0005890 |
| Patient date of birth | ADT | Measurement | Demographic factors | C0421451 |
| Patient sex | ADT | Measurement | Demographic factors | C0079399 |
| Weight | Consultation note | Measurement | Demographic factors | C0005910 |
| Arthrosis after infection | Consultation note | Problem | Diagnosis primary procedure | C0003869 |
| Aseptic necrosis | Consultation note | Problem | Diagnosis primary procedure | C0085660 |
| Fracture | Consultation note | Problem | Diagnosis primary procedure | C0016658 |
| Hip dysplasia | Consultation note | Problem | Diagnosis primary procedure | C1328407 |
| Inflammation | Consultation note | Problem | Diagnosis primary procedure | C0021368 |
| Posttraumatic arthrosis | Consultation note | Problem | Diagnosis primary procedure | C1388734 |
| Primary arthrosis | Consultation note | Problem | Diagnosis primary procedure | C1388735 |
| Primary procedure | Consultation note | Procedure | Diagnosis primary procedure | LC0006478 |
| Rheumatoid arthritis | Consultation note | Problem | Diagnosis primary procedure | C0003873 |
| Secondary arthrosis | Consultation note | Problem | Diagnosis primary procedure | C2732281 |
| Tumor | Consultation note | Problem | Diagnosis primary procedure | C0027651 |
| Discharge from hospital | Discharge letter | Procedure | Hospitalization | C2361123 |
| Hospital admission | Hospitalization note | Procedure | Hospitalization | C0184666 |
| Readmission | Hospitalization note | Procedure | Hospitalization | C0030700 |
| Anesthesia | Surgery note | Measurement | Inpatient procedure | C1305863 |
| Cerclage | Surgery note | Procedure | Inpatient procedure | C1292825 |
| Diagnosis at surgery | Surgery note | Measurement | Inpatient procedure | C2315323 |
| Hip approach | Surgery note | Measurement | Inpatient procedure | C0449445 |
| Insertion of therapeutic spacer into joint | Surgery note | Procedure | Inpatient procedure | C1960345 |
| Nomenclature | Surgery note | Measurement | Inpatient procedure | C4019166 |
| Psoas release | Surgery note | Procedure | Inpatient procedure | LC0006468 |
| Reduction | Surgery note | Procedure | Inpatient procedure | C1293152 |
| Wound drainage | Surgery note | Procedure | Inpatient procedure | C0920263 |
| Wound wash | Surgery note | Procedure | Inpatient procedure | C0455099 |
| Hip joint - range of motion | Consultation note | Measurement | Joint specific findings | C0080078 |
| Hip joint laxity | Consultation note | Measurement | Joint specific findings | C0086437 |
| Hip joint stability | Consultation note | Measurement | Joint specific findings | C0427257 |
| Mobility | Consultation note | Measurement | Joint specific findings | C0425245 |
| Antibiotics | Drug exposure | Medication | Medication exposure | C0003232 |
| Antithrombotics | Drug exposure | Medication | Medication exposure | C1704311 |
| NSAID | Drug exposure | Medication | Medication exposure | C0003211 |
| Pain medication | Drug exposure | Medication | Medication exposure | C0002771 |
| Pregabalin | Drug exposure | Medication | Medication exposure | C0657912 |
| Death | Consultation note | Problem | Other | C0011065 |
| Allograft surgical material | Surgery note | Equipment | Other equipment | C0450127 |
| Operative video system | Surgery note | Equipment | Other equipment | C0589547 |
| Total hip replacement prosthesis | Surgery note | Equipment | Other equipment | C0302751 |
| Usage of bone substitute | Surgery note | Equipment | Other equipment | C0243003 |
| Leg length difference | Consultation note | Measurement | Other findings | C0426952 |
| LOS (Length of Stay) | Consultation note | Measurement | Other findings | C0023303 |
| Scar status | Consultation note | Measurement | Other findings | C2711903 |
| Aspiration | Surgery note | Procedure | Outpatient procedure | C0349707 |
| Blood transfusion | Surgery note | Procedure | Outpatient procedure | C0005841 |
| Puncture | Surgery note | Procedure | Outpatient procedure | C0034117 |
| Corticoid infiltration | Surgery note | Procedure | Procedure | LC0040128 |
| Cortisone infiltration | Surgery note | Procedure | Procedure | C0702249 |
| Hyaluron infiltration | Surgery note | Procedure | Procedure | LC0012345 |
| Infiltration | Surgery note | Procedure | Procedure | C0702249 |
| Infiltration psoas | Surgery note | Procedure | Procedure | LC0040125 |
| Intra-articular infiltration | Surgery note | Procedure | Procedure | C0021488 |
| Intramuscular infiltration | Surgery note | Procedure | Procedure | C0394862 |
| Marcainization | Surgery note | Procedure | Procedure | LC0040126 |
| Physiotherapy | Surgery note | Procedure | Procedure | C0949766 |
| Date of admission | Surgery note | Measurement | Procedure specific | C1302393 |
| Date of procedure | Surgery note | Measurement | Procedure specific | C2584899 |
| Primary hip arthroplasty | Surgery note | Procedure | Procedure specific | C3472555 |
| Revision of hip arthroplasty | Surgery note | Procedure | Procedure specific | C0186201 |
| Revision technique | Surgery note | Procedure | Procedure specific | C1527075 |
| Surgery date | Surgery note | Measurement | Procedure specific | C4255413 |
| Surgery duration | Surgery note | Measurement | Procedure specific | C1442476 |
| Total hip replacement | Surgery note | Procedure | Procedure specific | C0040508 |
| Configuration | Surgery note | Measurement | Prosthesis equipment | LC0040071 |
| Cup brand | Surgery note | Measurement | Prosthesis equipment | LC0017603 |
| Cup manufacturer | Surgery note | Measurement | Prosthesis equipment | C3843886 |
| Cup size | Surgery note | Measurement | Prosthesis equipment | LC0017502 |
| Cup type | Surgery note | Measurement | Prosthesis equipment | LC0014219 |
| Greffen | Surgery note | Measurement | Prosthesis equipment | C0449401 |
| Hip Joint Implantable Prosthesis (device) | Surgery note | Equipment | Prosthesis equipment | C0019560 |
| Insert brand (liner) | Surgery note | Measurement | Prosthesis equipment | LC0040103 |
| Insert manufacturer (liner) | Surgery note | Measurement | Prosthesis equipment | LC0040102 |
| Insert size (liner) | Surgery note | Measurement | Prosthesis equipment | LC0008004 |
| Insert type (liner) | Surgery note | Measurement | Prosthesis equipment | LC0014734 |
| Head diameter | Surgery note | Measurement | Prosthesis equipment | LC0013523 |
| Head brand (femoral head) | Surgery note | Measurement | Prosthesis equipment | LC0040067 |
| Head manufacturer (femoral head) | Surgery note | Measurement | Prosthesis equipment | LC0018301 |
| Head size (femoral head) | Surgery note | Measurement | Prosthesis equipment | LC0017503 |
| Head type (femoral head) | Surgery note | Measurement | Prosthesis equipment | LC5500004 |
| Prosthesis manufacturer | Surgery note | Measurement | Prosthesis equipment | C3843886 |
| Prosthesis size | Surgery note | Measurement | Prosthesis equipment | LC0014406 |
| Steel brand (stem) | Surgery note | Measurement | Prosthesis equipment | LC0040064 |
| Steel manufacturer (stem) | Surgery note | Measurement | Prosthesis equipment | LC0040065 |
| Steel size (stem) | Surgery note | Measurement | Prosthesis equipment | LC0017504 |
| Steel type (stem) | Surgery note | Measurement | Prosthesis equipment | LC0040068 |
| Anesthesiologist | Surgery note | Historical info | Surgical team | C0334910 |
| Assistant | Surgery note | Historical info | Surgical team | C0031833 |
| Surgeon | Surgery note | Historical info | Surgical team | C0582175 |
| Avascular necrosis | Consultation note | Problem | Symptoms | C3887513 |
| Fracture | Consultation note | Problem | Symptoms | C0016658 |
| Limited ROM | Consultation note | Measurement | Symptoms | C0231589 |
| Limited walking distance | Consultation note | Measurement | Symptoms | C0424550 |
| Morning stiffness | Consultation note | Problem | Symptoms | C0856842 |
| Osteoarthritis | Consultation note | Problem | Symptoms | C1384584 |
| Pain | Consultation note | Problem | Symptoms | C0030193 |
| Radiating pain | Consultation note | Problem | Symptoms | C0234254 |
